# Gastrointestinal helminths of Coyotes (*Canis latrans*) from Southeast Nebraska and Shenandoah area of Iowa

**DOI:** 10.14202/vetworld.2016.970-975

**Published:** 2016-09-15

**Authors:** Whitni K. Redman, Jay E. Bryant, Gul Ahmad

**Affiliations:** Department of Natural Sciences, School of Arts & Sciences, Peru State College, Peru Nebraska 68421-0010, USA

**Keywords:** cestode, coyotes, helminths, infection, intestinal, nematode

## Abstract

**Aim:**

This survey was carried out on the carcasses of 29 coyotes from Southeastern Nebraska and Shenandoah area of Iowa to document the helminths present in the intestinal track of these carnivorous animals.

**Materials and Methods:**

A total of 29 adult coyote carcasses were generously donated in the autumn and winter (November-February) of 2014-2015 by trappers, fur buyers and hunters of Southeast Nebraska and Shenandoah area of Iowa. The intestine of individual animals were examined for the recovery of helminth parasites as per the established procedures.

**Results:**

We found that as many as 93.10% of the investigated coyotes were infected with one or more helminth infections. A total of 10 different species of helminth parasites were recovered from the intestines of coyotes under investigation. Among the 10 species of helminths, 5 were identified as cestodes while the remaining 5 were nematodes. A total of 82.75% of the animals were infected with one or more species of nematodes, while 75.86% of them were colonized with one or more species of cestode parasites. The most abundant species in coyotes were *Toxascaris leonina* (68.95%) closely followed by *Taenia hydatigena* (58.62%). The prevalence of *Ancylostoma caninum* and *Taenia pisiformis* were recorded at 31.03%, followed by those of *Toxocara canis* and *Echinococcus* spp. at 24.13%, respectively. Three animals were infected with *Trichuris vulpis* while three other coyotes each were found to be harboring *Uncinaria stenocephala*, *Dipylidium caninum*, or *Hymenolepis diminuta*. The presence of *H. diminuta* might have been the result of the ingestion of a rodent by the respective coyotes.

**Conclusion:**

From the overall analysis of the present data and comparing it with the previous reports of various scientists over several decades, we can conclude that intestinal helminths are still very much prevalent among the coyote population in the Southeast Nebraska and Iowa area. The relatively high prevalence of the zoonotic parasite species further warrants a more comprehensive investigation with larger numbers of wild predators from the region to ascertain the possible contribution of coyotes to the disease cycle as these animals are more frequently spotted in and around the densely populated urban areas.

## Introduction

As more and more coyotes have been spotted in and around the major urban areas of the United States, particularly in the Midwest region of our country, they are bound to have close contact with human populations, thereby impacting public health by increasing the chances of transmitting some zoonotic parasites to man. The potential health hazards related to the transmission of zoonotic diseases are echinococcosis, taeniasis, toxocariasis, leishmaniasis, rabies, Chagas disease, plague, and other microbial infections. It’s reasonable to assume that these animals are moving to metropolitan areas because their habitats have been invaded by humans; other displacement factors include climate change and habitat destruction. Most of the time these invading coyotes keep a safe distance from human beings, but the conflict is inevitable when they are forced to share limited space. Often, tussles arise between invading predators and domesticated animals. As the wild carnivores (i.e., coyotes) and domesticated dogs are in close proximity in terms of their habitat, it would be imperative to investigate the prevalence in parasitic infections among these animals. Some investigations were conducted by Canadian scientists on the prevalence and intensity of gastrointestinal helminths of wild predators, including coyotes, both from the rural forested areas, as well as some metropolitan regions of that country [1-6].

A number of direct or indirect studies have been previously reported from the United States including the states of New York, Wyoming, Kansas, Illinois, Iowa, Utah, and Tennessee on the prevalence of gastrointestinal parasites of wild predators including coyotes [7-12]. The study from New York regions [7] examined 145 fecal samples of coyotes in the years 2000-2001 and identified several parasites including protozoa, helminths, and arthropodes. These reports identified, a total of 12 different species of helminths based on the microscopic examinations of the fecal materials. In a related study, the gastrointestinal content of 144 coyotes from Iowa were examined, out of which 141 or 97.9% of these animals were infected with one or more species of helminth parasites. The most abundant among the helminth parasites were those of *Taenia* species [11]. However, limited information is available on the gastrointestinal parasites of coyotes from the Southeastern region of Nebraska. A study was carried out more than two decades ago [13] on the prevalence of heartworm - *Dirofilaria immitis* among the domestic and wild canids of the Southeastern Nebraska. These workers found that 21.4% of *Canis familiaris* (domestic dogs) and 8.9% of *Canis latrans* coyotes were infected with heartworm. Unfortunately, the above-mentioned investigators limited their study to the documentation of *D. immitis* alone and neglected the prevalence of gastrointestinal parasites of these carnivores. Another group [8] examined 30 coyotes from Southern Nebraska for the prevalence of *Echinococcus multilocularis* and attributed the absence of *E. multilocularis* in wild canids to the absence of the parasite in these areas (Southern Nebraska, Kansas) or a sufficiently low prevalence (1-2% in canids) so that it was not detected based on the number of hosts examined.

The aims of the present investigation were to determine the prevalence and intensity of gastrointestinal helminth parasites of coyotes from the Southeastern region of Nebraska and Shenandoah area of Iowa.

## Materials and Methods

### Ethical approval

This study was approved by Institutional Review Board of Peru State College (PSC IRB).

### Necropsy of coyotes; isolation and identification of parasites

A total of 29 adult coyote carcasses were generously donated in the autumn and winter (November-February) of 2014-2015 by trappers, fur buyers and hunters of Southeast Nebraska and Shenandoah area of Iowa. Data on individual coyotes were collected (trapping location, date, sex, and age). Sexes of the animals (17 males and 12 females) were determined by gross examination. Carcasses were refrigerated or frozen until necropsy. A detailed necropsy of each coyote was performed after removing the individual organs. Visual examinations of each organ were done by two different individuals to ensure that all the prevalent parasites were recovered. Intestines of each animal were collected at necropsy and refrozen at −20°C. The small and large intestines were opened longitudinally, and the contents of these organs were emptied in a 2 L large beaker. The intestinal contents were washed with tap water, and the sediments were allowed to settle for 15 min. The clear supernatants were filtered into another beaker, and again the small particulates/eggs were allowed to settle at the bottom of the container. The supernatants from these filtrates were discarded, saving the sediments to be examined both macroscopically and microscopically for the intestinal parasites and their eggs. Based on gross structure and location, helminths were separated into groups and counted. Individual worms were collected and washed several times before being preserved in 70% ethanol for further identifications and characterization. The identification of individual nematode species is done under the microscope at 100× to 400× magnification [14]. Identification of cestode species was performed by a measurement of large and small hooks as well as blade: Handle ratios [15].

### Statistical analysis

Statistical analysis was performed using SPSS program (version 17.0 package, IBM Corporation, Armonk, New York, USA).

## Results

**Figure 1:**
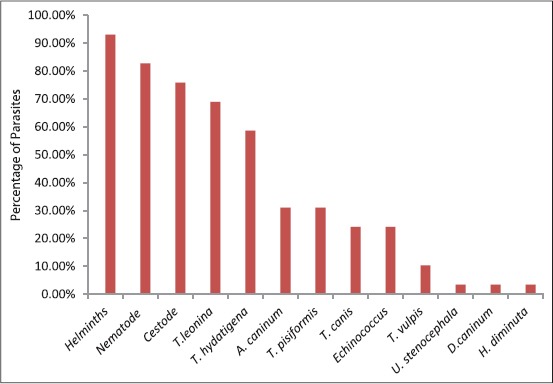
Percent abundance of individual helminth parasite recovered from the intestine of coyotes at postmortem examination of carcasses.

The results of the present investigation are summarized in Tables-1 and 2. As can be seen from the content of Table-1, of 29 coyotes investigated, 17 were male (58.62%) and 12 were female (41.37%). The number of different species of helminths recovered also varied depending on the individual infected animal. As many as 175 *Taenia hydatigena* were recovered from coyote number 3 making it the most heavily infected animal with this cestode parasite in the current study. Coyote number 1 had 58 *Toxascaris leonina*, making it the highest number of nematode species recovered from an individual animal under investigation. The details of the type of helminths and the number of each parasite recovered from the individual host is given in Table-1 in parenthesis. As can be shown from the Table-2, 27 of the 29 animals dissected, in this study, were infected with one or more helminth infections, which brings the percent infection rate of parasitic helminths to a whopping 93.1%. Among the parasites recovered from these coyotes, nematodes were 82.75%, while the recovery of cestodes stood very closely at 75.86%. In terms of the kinds of parasites present at the species level, the most abundant parasite was *Toxascaris leonina*, which was recovered from 20 of 29 coyotes making the percent abundance of this parasite 68.95%. The next dominant parasite in the intestines of coyotes was *T. hydatigena*, which was found in 17 of the 29 coyotes and the percent abundance of it was calculated to be 58.62%. One-third of the parasites that infected the coyotes were the hookworm *Ancylostoma caninum* and the cestode parasite - *Taenia pisiformis*. The percentage of coyotes bearing these two helminths was 31.03%. The next two abundant parasites were *Toxocara canis* and *Echinococcus* spp. which infected about a quarter of the examined coyotes in this study. The percent abundance of these two helminths stood at 24.13% as these parasites were recovered from 7 of 29 animals. Three of the *Canis latrans* examined harbored *Trichuris vulpis*, bringing the percent abundance of the whip worm to 10.34%. In addition to the above-mentioned nematode and cestodes, three more helminths were recovered from only one of the 29 animals under study. These helminths were *Unicinaria stenocephala*, *Dipylidium caninum*, and *Hymenolepis diminuta*. In terms of the number of species, a total of five different species of nematodes and five species of cestodes were recovered from the intestines of coyotes in the present investigations. The percent abundance of groups as well as individual helminth parasite species recovered from the intestine of coyotes at postmortem examination of carcasses is depicted in Figure-1.

**Table-1:** The number and type of helminth parasites recovered from each coyotes dissected in the present investigation.

Coyote#	Coyote sex	Total number of nematode	Total number of cestode	Species of nematode	Species of cestode
COY1	M	58	32	*T. leonina* (58)	*T. hydatigena* (32)
COY2	M	38	18	*T. leonina* (28) *A. caninum* (8)	*T. hydatigena* (15) *T. pisiformis* (3)
COY3	F	1	190	*T. leonina* (1)	*T. hydatigena* (175)
					*E. multilocularis* (8)
					*T. canis* (7)
COY4	M	1	22	*T. leonina* (1)	*T. hydatigena* (15) *D. caninum* (7)
COY5	F	4	0	*T. leonina* (4)	0
COY6	F	1	13	*A. caninum* (1)	*T. hydatigena* (13)
COY7	M	9	0	*T. leonina* (5)	0
				*A. caninum* (2)	
				*T. canis* (2)	
COY8	M	49	182	*T. leonina* (9)	*E. multilocularis* (162)
				*A. caninum* (17)	
				*T. canis* (8)	
				*T. vulpis* (3)	
				*U. stenocephala* (12)	
COY9	M	51	0	*T. leonina* (5) *A. caninum* (46)	0
COY10	F	18	102	*T. leonina* (13)	*T. hydatigena* (86)
				*A. caninum* (5)	*T. pisiformis* (16)
COY11	M	3	9	*T. leonina* (3)	*T. hydatigena* (9)
COY12	M	15	53	*T. leonina* (9)	*T. hydatigena* (35)
				*A. caninum* (4)	*T. pisiformis* (18)
				*T. canis* (2)	
COY13	F	5	22	*T. leonina* (9)	*T. hydatigena* (35)
COY14	M	6	0	*T. canis* (2)	0
COY15	F	0	154	0	*T. hydatigena* (32)
					*T. pisiformis* (83)
					*E. multilocularis* (39)
COY16	F	0	247	0	*T. hydatigena* (76)
					*T. pisiformis* (32)
					*E. multilocularis* (139)
COY17	M	1	50	0	*T. hydatigena* (32) *T. pisiformis* (18)
COY18	M	30	0	*T. leonina* (30)	0
COY19	F	8	0	*T. leonina* (5)	0
				*A. caninum* (2)	
				*T. vulpis* (1)	
COY20	M	6	89	*T. leonina* (6)	*T. hydatigena* (89)
COY21	F	17	11	*T. leonina* (15)	*H. diminuta* (11)
				*T. vulpis* (2)	
COY22	M	0	0	0	0
COY23	F	18	59	*T. leonina* (18)	*T. hydatigena* (59)
COY24	M	12	12	*T. canis* (1)	*T. hydatigena* (12)
					*T. pisiformis* (12)
COY25	M	1	74	*T. canis* (1)	*T. hydatigena* (31)
					*T. pisiformis* (40)
					*E. multilocularis* (3)
COY26	F	1	3	*T. leonina* (1)	*T. hydatigena* (1)
					*E. multilocularis* (2)
COY27	M	1	8	*T. leonina* (1)	*T. pisiformis* (8)
COY28	F	13	17	*T. leonina* (1)	*E. multilocularis* (17)
				*A. caninum* (12)	
COY29	M	0	0	0	0

The number in the parenthesis indicates the number of individual parasites recovered from that animal. *T. leonina=Toxascaris leonina, A. caninum=Ancylostoma caninum, T. canis=Toxocara canis, T. vulpis=Trichuris vulpis, U. stenocephala=Uncinaria stenocephala, T. hydatigena=Taenia hydatigena, T. pisiformis=Taenia pisiformis, E. multilocularis=Echinococcus multilocularis, D. caninum=Dipylidium caninum, H. diminuta=Hymenolepis diminuta*

**Table-2:** The percent infection of coyotes with various helminths parasites.

Parasites	Number of coyotes infected	Percent abundance
Helminths	27/29	93.10
Nematodes	24/29	82.75
Cestodes	22/29	75.86
*T. leonina*	20/29	68.95
*T. hydatigena*	17/29	58.62
*A. caninum*	9/29	31.03
*T. pisiformis*	9/29	31.03
*T. canis*	7/29	24.13
*Echinococcus* sp.	7/29	24.13
*T. vulpis*	3/29	10.34
*U. stenocephala*	1/29	3.44
*D. caninum*	1/29	3.44
*H. diminuta*	1/29	3.44

*T. leonina=Toxascaris leonina, A. caninum=Ancylostoma caninum, T. canis=Toxocara canis, T. vulpis=Trichuris vulpis, U. stenocephala=Uncinaria stenocephala, T. hydatigena=Taenia hydatigena, T. pisiformis=Taenia pisiformis, E. multilocularis=Echinococcus multilocularis, D. caninum=Dipylidium caninum, H. dimunita=Hymenolepis diminuta*.

## Discussion

In the present investigation of the 29 coyotes examined, 27 were found to be infected with one or more species of helminths. It was observed that seven of the animals under investigation were free of any cestode parasites while only four of the coyotes were free of the nematode. COY15, COY16, COY17 specifically harbored cestode parasites. On the other hand, the parasites recovered from COY7, COY9, COY14, COY18, and COY19 belonged to the phylum nematode. The number of parasites recovered from the individual animals also varied, ranging from a low of 4 helminths in COY5 to a high of 247 in COY16. Overall, analysis of our results indicates that the prevalence of helminths among coyotes is 93.10% which is somewhat lower compared with the reported data in the literature from the region [10]. These researchers reported a consistently high prevalence of helminths among coyotes to the tune of 97.9% of the animals. Overall, the occurrence and prevalence of helminth species that we reported here for coyotes in Southeast Nebraska and Shenandoah area of Iowa are similar to what was previously observed for the parasites of coyotes from the state of Montana, USA [16]. We identified 10 different species of helminth parasites in our study while others have reported as many as 19 species of parasites from coyotes based on fecal examination using sugar and zinc flotation techniques [7]. Since the specific gravity of zinc flotation solution has a direct impact on the recovery of helminth eggs, specifically those of trematode parasite eggs, therefore a lower number of parasite species in this study makes sense as our report is based on the recovery of actual number of adult parasites from the gastrointestinal organ of the animals. For example, we found 7 of the 29 coyotes harboring *T. canis* which translates to 24.13% abundance of this parasite in coyote populations but others using noninvasive fecal sample examinations reported either higher number of parasites species or a very low abundance of this species depending on the kind of specific gravity of the floating solution used by the researchers [1,17-20]. The present investigation is in agreement with the previous report [10] in terms of the prevalence of various species of *Taenia* among the coyote populations in Southeast Nebraska. Among the cestode parasites, *T. hydatigena* was the dominant species, infecting more than 58% of the animals under investigation. The highest prevalence of helminth parasites in our study was *T. leonina*, which stood at 68.95% as 20 of the 29 coyotes examined harbored this particular nematode parasite. This finding concurs with the work of previous scientists who have reported the highest prevalence of *T. leonina* among the 25 coyotes which they examined [17]. Although the prevalence of *T. leonina* was also reported to be relatively low among the various localities of Tennessee [12]. More recently [21] a very low prevalence of *T. leonina* among the coyotes from Newfoundland region of Canada was reported.

Among the other *Taenia spp*. which moderately colonized the intestine of coyotes found in the present study was *T. pisiformis* the prevalence of which was 31% with an intensity of infection ranging from 12 parasites in COY24 to 83 parasites in COY15. This parasite was previously reported to be common among coyotes where the intensity of infection ranged from a low of 43 to a high of 83 parasites with a prevalence rate of 63.9% [12]. However, more recently [21] reported a very low prevalence of *T. pisiformis* in Newfoundland area of Canada where they found just 1% of coyotes infected with this cestode parasite. We also found that 24.13% of the examined coyote carcasses were infected with *E*. *multilocularis* parasite. The intensity of echinococcosis ranged from a very low range of 2 parasites in COY26 to 162 parasites in COY8. Previously others [8] reported a lower prevalence of *E. multilocularis* in wild canids in western Nebraska and Wyoming compared to northeastern Nebraska, in that only 2 of 42 (4.8%) red foxes from the west of these states were infected compared to 25 of 56 (44.6%) red foxes from the northeast. Some authors [22] attributed the spread of *E. multilocularis* to the larger home ranges and longer traveling distance of coyotes as compared to red foxes. Unfortunately, these researchers could not assess the pattern of infection among the coyote population, but assumed the prevalence of this zoonotic parasite would be similar to those of the red foxes. We found 7 of the 29 coyotes (24.13%) to be infected with *E. multilocularis* in Southeast Nebraska which makes the assumption of the previous workers a reality. It is becoming quite dangerous as more and more populations of wild canids including coyotes are found in urban areas where the density of human populations is high [23]. As a result of the present data, it is a very important to monitor the distribution, prevalence and spread of this zoonotically important parasite in various areas of the Midwest as environmental fecal contaminations by coyotes may lead to infection of small rodent populations in and around metropolitan areas, leading to infection of domestic dogs that occasionally prey on these small mammals and thereby become a potential source of human infection [24].

*Ancylostoma caninum* was another parasite which was detected in almost one-third of the coyotes examined in the current study. The intensity of infection with these pathogenic parasites ranged from a low of 1 parasite in COY6 to a high of 46 worms in COY9 animal. Previously others also detected a similar prevalence of *A. caninum* among the coyote population of Iowa [10]. While another group [7] reported a very low prevalence of *Uncinaria stenocephala* in two of the three sites examined. We also found a similarly low prevalence (3.44%) for this hookworm parasite which is in agreement with others work [7]. In the past, investigators considered uncinariasis less pathogenic as compared with the more pathogenic sister species, *A. caninum* [25], but some authors [26] observed both of these species in Eastern coyotes as north as southern Pennsylvania.

We found just over 10% of the animals were infected with *T. vulpus*; as a result, we can concur that the trend in the prevalence of *T. vulpus* has not changed over time in the region. Based on their study of 144 coyotes [10] found the less frequent presence of the *T. vulpis* parasite in Iowa coyotes, and observed the infection of this parasite primarily in younger animals. On the other hand, different studies [12] found a very high prevalence of *T. vulpis* (55.6%) among the coyotes from 30 different localities of Tennessee and attributed this unusually high prevalence and intensity of *T. vulpis* to the climate of the region. Many other studies previously also associated the high prevalence of this parasite to the abundance of moisture, dense vegetation, and heavy rainfalls [27,28]. Of 29 coyote carcasses examined in this study almost all of them were found to be infected with one or more species of cestode parasites. These findings indicate that there is not much change in terms of prevalence and diversity of the helminth parasite of coyotes for the past 40 years [10]. The presence of *Hymenolepis diminuta* in one of the 29 coyotes was surprising, but previously others also reported this dwarf cestode parasite in coyotes and attributed their presence to a possible spurious parasite resulting from the ingestion of rodents by coyotes [29].

These results indicate that *C. latrans* harbor a number of parasitic helminths in their gastrointestinal tract including but not limited to a number of zoonotic parasites which could have a potential role to play in the prevalence of these parasites in the urban and semi-urban areas. As a result, coyotes could be an important potential reservoir for transmission of the disease to domestic dogs as well as human, which act as intermediate hosts to some of the parasites like *Echinococcus* species. From the overall analysis of the present data and comparing it with the previous reports of various scientists over several decades, we can conclude that intestinal helminths are still very much prevalent among the coyote population in the Southeast Nebraska and Iowa area. The relatively high prevalence of the zoonotic parasite species further warrants a more comprehensive investigation with larger numbers of wild predators from the region to ascertain the possible contribution of coyotes to the disease cycle as these animals are more frequently spotted in and around the densely populated urban areas.

## Authors’ Contributions

W.K.R, J.E.B and G.A, all were equally contributed in the collection, storage and freezing of the coyotes carcasses. Design of the experimental protocol and technical help in necropsy of the animals was the job of GA. Dissection of coyote’s gastrointestinal organs and isolations of the helminth parasites was the work of WKR and JEB. Identification of individual parasites: GA. Draft of the manuscript by WKR and JEB under the supervision of GA. Correction of the manuscript and data analysis: GA. All the authors read and approved the final version of the manuscript.
